# FUS Recognizes G Quadruplex Structures Within Neuronal mRNAs

**DOI:** 10.3389/fmolb.2020.00006

**Published:** 2020-02-07

**Authors:** Joshua A. Imperatore, Damian S. McAninch, Arielle N. Valdez-Sinon, Gary J. Bassell, Mihaela Rita Mihailescu

**Affiliations:** ^1^Department of Chemistry and Biochemistry, Duquesne University, Pittsburgh, PA, United States; ^2^Department of Cell Biology, Emory University School of Medicine, Atlanta, GA, United States

**Keywords:** ALS, FTD, FUS, G quadruplex RNA, RGG box, mRNA

## Abstract

Fused in sarcoma (FUS), identified as the heterogeneous nuclear ribonuclear protein P2, is expressed in neuronal and non-neuronal tissue, and among other functions, has been implicated in messenger RNA (mRNA) transport and possibly local translation regulation. Although FUS is mainly localized to the nucleus, in the neurons FUS has also been shown to localize to the post-synaptic density, as well as to the pre-synapse. Additionally, the FUS deletion in cultured hippocampal cells results in abnormal spine and dendrite morphology. Thus, FUS may play a role in synaptic function regulation, mRNA localization, and local translation. Many dendritic mRNAs have been shown to form G quadruplex structures in their 3′-untranslated region (3′-UTR). Since FUS contains three arginine-glycine-glycine (RGG) boxes, an RNA binding domain shown to bind with high affinity and specificity to RNA G quadruplex structures, in this study we hypothesized that FUS recognizes these structural elements in its neuronal mRNA targets. Two neuronal mRNAs found in the pre- and post-synapse are the post-synaptic density protein 95 (PSD-95) and Shank1 mRNAs, which encode for proteins involved in synaptic plasticity, maintenance, and function. These mRNAs have been shown to form 3′-UTR G quadruplex structures and were also enriched in FUS hydrogels. In this study, we used native gel electrophoresis and steady-state fluorescence spectroscopy to demonstrate specific nanomolar binding of the FUS C-terminal RGG box and of full-length FUS to the RNA G quadruplex structures formed in the 3′-UTR of PSD-95 and Shank1a mRNAs. These results point toward a novel mechanism by which FUS targets neuronal mRNA and given that these PSD-95 and Shank1 3′-UTR G quadruplex structures are also targeted by the fragile X mental retardation protein (FMRP), they raise the possibility that FUS and FMRP might work together to regulate the translation of these neuronal mRNA targets.

## Introduction

**Figure 1:**
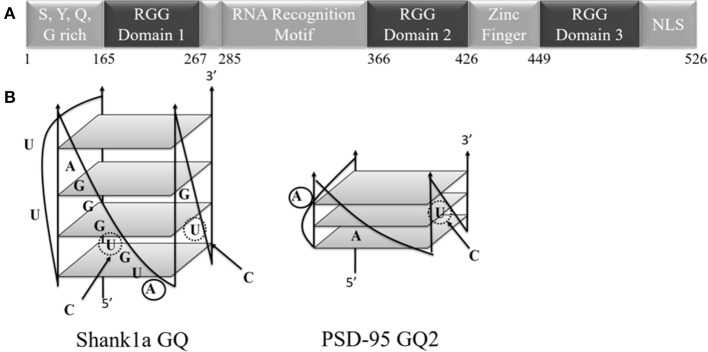
**(A)** Schematic diagram of the FUS domains showing the SYQG rich region, the RNA recognition motif and three arginine-glycine-glycine (RGG) domains. **(B)** Predicted structure of Shank1a GQ and PSD-95 GQ2 (Stefanovic et al., 2014; Zhang et al., 2014). C to U mutations indicated with arrows and dashed circles, and 2-AP substitutions are denoted by solid circles.

Fused in sarcoma (FUS), identified as the heterogeneous nuclear ribonuclear protein P2, belongs to the family of proteins consisting of FUS, Ewing's Sarcoma, and TATA-binding protein associated factor 15 (FET). The FET family is predominantly localized to the nucleus and highly expressed in all examined tissues (Zinsner et al., 1994; Morohoshi et al., 1996; Andersson et al., 2008). Each of the FET proteins contains an RNA recognition motif (RRM), arginine-glycine-glycine repeat (RGG) regions, and a Cys_2_-Cys_2_ zinc finger (Iko et al., 2004; Tan and Manley, 2009). Specifically, FUS has a low-complexity prion like domain, three RGG regions, an N-terminal region with transcriptional activating properties, and a C-terminal region capable of binding DNA, RNA, and splicing factors (Figure 1; Law et al., 2006). Nuclear FUS shuttling is facilitated by its C-terminal proline-tyrosine nuclear localization signal (NLS) and methylation of its C-terminal RGG domain (RGG3 domain; Lee et al., 2006; Dormann et al., 2012). Mutations in FUS are associated with familial amyotrophic lateral sclerosis (ALS), as well as with sporadic ALS, with many of these mutations being localized in the protein C-terminus within its RGG3 domain (Kwiatkowski et al., 2009; Vance et al., 2009; Dejesus-Hernandez et al., 2010; Lai et al., 2011). In a human genome-wide approach, more than 5,500 *in vivo* RNA targets were identified for FUS (Lagier-Tourenne et al., 2012). FUS can target secondary structural elements such as hairpin structures with UU or UC pairing at the base of the loop (Hoell et al., 2011), G quadruplex (GQ) forming human telomere DNA, and a telomeric repeat containing RNA (Takahama et al., 2013). These DNA GQ structures are targeted by the FUS RGG3 domain (Takahama et al., 2013), which should be folded into a β-spiral structure for efficient binding (Ryota et al., 2018). GQ structures are formed when four guanine residues assemble into a planar G-quartet through Hoogsteen base pairing, stabilized by a central potassium ion, with several of these G quartet stacks forming a GQ (Sen and Gilbert, 1990; Hud et al., 1996).

Transcription and mRNA processing are among the many nuclear functions of FUS (Calvio et al., 1995; Aman et al., 1996; Tan and Manley, 2009; Lagier-Tourenne et al., 2010), however, studies have revealed that FUS functions outside of the nucleus as well. FUS has been observed within neutrophil granules (Aoki et al., 2012), clustered in the post-synaptic density of rat hippocampus and co-localized with marker SYP1 (Schoen et al., 2016). It has also been shown that in the early stages of synapse development, FUS is postsynaptically localized both in rodent synapses, and in human motoneurons derived from a healthy control induced pluripotent stem cells (Deshpande et al., 2019). Mouse studies suggest that synapses are significantly more susceptible to defects than axons and cell bodies when FUS is overexpressed or mutated (Sephton et al., 2016), indicating that FUS is important for synaptic plasticity and maintenance. mGluR activation causes the translocation of FUS to the dendrite (Fujii et al., 2005), strongly suggesting a role as a synaptic RNA-binding protein for mRNA transport and translation regulation at the dendrite (Fujii et al., 2005; Liu-Yesucevitz et al., 2011; Aoki et al., 2012). This would affect synaptic plasticity and maintenance, which may explain abnormal spine density and morphology in FUS knockdown mice (Fujii et al., 2005; Sephton et al., 2016).

Translation regulation can often be facilitated in RNA granules, where mRNA can be stored for transport to specific cytoplasmic regions, such as the dendrite, where it can be locally translated in response to synaptic input (Mahowald, 1962; Knowles et al., 1996). Hydrogel droplets composed of the low complexity (LC) prion-like domain of FUS were generated to mimic the formation and dissasembly of FUS-containing RNA granules. The FUS LC domain hydrogels have been shown to trap GFP-linked LC FUS (homotypic trapping), as well as the GFP-linked LC domains of 10 other RNA-binding proteins tested including FMRP, and the TAR DNA-binding protein 43 (TDP-43), etc (heterotypic trapping) via LC-LC domains interactions (Kato et al., 2012). Moreover, FUS LC domain hydrogels incubated with mouse brain cell or human U2OS cell lysates trapped mRNAs that are proposed to be targeted to the granules by LC-domain containing RNA-binding proteins, including endogenous FUS, that interact via their LC-LC domains (Han et al., 2012). Among these trapped mRNAs, 11 mRNAs, thought to be normally part of neuronal granules to be transported to the denrites, were enriched. Interestingly, seven of these 11 mRNAs that enter neuronal granules are either predicted or shown to form GQ structures in their 3′-UTRs (Subramanian et al., 2011; Stefanovic et al., 2014; Zhang et al., 2014).

Thus, given FUS's regulatory role at the synapse and the fact that it contains RNA-binding domains that have been shown to bind a telomeric RNA GQ structure, we hypothesized that FUS could also recognize GQ structures in the 3′UTR of some of its neuronal mRNA targets. To test this hypothesis we selected two neuronal mRNAs found enriched in the FUS LC hydrogels which encode for protein components of the post synaptic density Shank1 and PSD-95, and which we previously showed form GQ structures in their 3′ UTRs (Stefanovic et al., 2014; Zhang et al., 2014). Shank1 mRNA was identified as a transcript targeted by FUS (Nakaya et al., 2013) and the PSD-95 protein levels have been shown to be reduced by FUS depletion in synaptoneurosomes (Udagawa et al., 2015). Shank1 and PSD-95 mRNAs are also targets of FMRP, whose loss of expression leads to the fragile X syndrome (Schütt et al., 2009; Muddashetty et al., 2011; Stefanovic et al., 2014; Zhang et al., 2014). Interestingly, Sephton et al. notes that FUS regulation at the synapse somewhat parallels FMRP's (Sephton et al., 2016). FMRP, like FUS, responds to mGluR activation and affects dendritic spine shape by regulating local translation of mRNAs at the synapse (Darnell and Klann, 2013). FMRP uses its RGG domain to bind GQ structures formed in mRNA targets (Darnell et al., 2001; Evans and Mihailescu, 2012; Blice-Baum and Mihailescu, 2014; Stefanovic et al., 2014, 2015; Zhang et al., 2014).

In this study, we found that FUS also binds the GQ structures formed in the Shank1 and PSD-95 mRNA 3′-UTRs with nanomolar dissociation constants. These results suggest a novel mechanism of GQ binding and mRNA regulation at the synapse by FUS, warranting additional studies exploring the roles of FUS and FMRP and of their possible cross-talk in the regulation of the transport, stability and translation of Shank1 or PSD-95 mRNAs.

## Materials and Methods

### RNA Synthesis

Wild-type PSD-95 GQ2, Shank1a GQ, their M1 mutants in which the GGUG sequence was disrupted but the GQ structure was preserved (mutations introduced showed in bold and italics in Table 1 and with dashed circles in Figure 1B) and the microtubule associated protein 1B (MAP1B) GQ were synthesized by *in vitro* transcription off synthetic DNA templates (Trilink Biotechnologies, Inc.) using T7 RNA polymerase produced in-house (Milligan and Uhlenbeck, 1989). Oligonucleotides were purified by 20% 8 M urea denaturing polyacrylamide gel electrophoresis (PAGE), electrophoretically eluted, and dialyzed against 10 mM cacodylic acid, pH 6.5. The mutants in which both, the GGUG sequence and the GQ structures were disrupted, PSD-95 M2 and Shank1a M2 (mutations introduced showed in bold and italics in Table 1), were chemically synthesized by Dharmacon, Inc. The highly fluorescent adenine analog, 2-aminopurine (2-AP), was used to replace the adenine at positions 18 and 4 (bolded in Table 1 and solid circles in Figure 1B) for PSD-95 GQ2 and Shank1a GQ mRNAs, respectively (Dharmacon, Inc.). The biotinylated PSD-95 Q1-Q2, Shank1a GQ, PSD-95 M2, and Shank1a M2 sequences were purchased from Dharmacon, Inc.

**Table 1:** RNA and peptide sequences used in this study.

Shank1a GQ	GGGG UU GGGG AGGG U GU**A** GGGG G U GGGG
Shank1a GQ M1	GGGG UU GGGG AGGG ***C*** GUA GGGG G ***C*** GGGG
Shank1a M2	G***CC***G UU G***CC***G AGGG ***C*** GUA G***CC***G G ***C*** G***CC***G
Shank1b GQ	GGGGAGGAGAGGUCGGGGUGGGGAGUGGGG
PSD-95 Q1	GGGAAAAGGGAGGGAUGGGUCUAGGG
PSD-95 GQ2	GGG **A** GGG A GGG U GGG
PSD-95 GQ2 M1	GGG A GGG A GGG ***C*** GGG
PSD-95 M2	G***C***G A G***C***G A G***C***G ***C*** G***C***G
PSD-95 Q1-Q2	GGGAAAAGGGAGGGAUGGGUCUAGGGAGUGGGAAAUGCGGG AGGGAGGGUGGG
FUS peptide	NH2-APKPDGPGGGPGGSHMGGNYGDDR **RGG RGG** YD **RGG** YRG **RGG** D **RGG** F **RGG RGG** GD **RGG** FGPGKMDSRGEHRQDRRERPY-CONH2

*Guanines involved in G quadruplex formation are underlined, fluorescent analogs are in bold for RNA sequences, and bold and italic letters indicate mutated nucleotides. RGG repeats are shown in bold for the FUS RGG3 peptide*.

### FUS Protein Expression and Purification

The plasmid encoding the full-length GST-fusion FUS and for the ALS-linked FUS R495X mutant, provided by Dr. Daryl Bosco from the University of Massachusetts Medical School, were transfected into Rosetta II DE3 cells (Novagen). All media used in cell growth consisted of Luria-Bertani (LB; Fisher Scientific) containing 200 μg/ml ampicillin (AMP; MP Biomedical) and 15 μg/ml chloramphenicol (CHL; MP Biomedical). A single colony was added to 5 ml of media and placed in a shaker at 225 rpm for 6 h at 37°C then added to 145 ml of media and incubated at 30°C overnight in a shaker at 225 rpm. Following overnight incubation, two 1 L cultures were generated by adding 50 ml of the overnight culture to each 4 L flask and were incubated at 20° C for 6–8 h. When the OD_600_ reached 0.8, 1 mM isopropyl β-D-1-thiogalactopyranoside (IPTG) and 5 mM ZnCl_2_ were added to each 1 L culture for induction and incubated for 22 h in a shaker at room temperature. The cells were harvested by centrifugation in 500 ml tubes, 4°C and stored overnight in the freezer. The cell pellets were thawed on ice and re-suspended in lysis buffer [30 ml of 50 mM Tris pH 8.0, 1 mM DTT, 0.1 mM EDTA, and protease inhibitor cocktail (Roche Diagnostics)] and 100 μg/ml RNase A. The samples were sonicated and then spun down at 13,000 rpm for 20 min at 4°C. The supernatant was added to 0.25 g glutathione-resin suspended in 30 ml of 50 mM Tris pH 8.0. This slurry was incubated on a rocker at 4°C for 2 h and then poured into a column, washed once with 10 ml of 50 mM Tris pH 8.0 and then eluted in five fractions by adding 15 ml of freshly prepared elution buffer (10 mM L-Glutathione, 1 mM DTT, 500 mM Tris, pH 9.5) to the column. The FUS wild type and FUS 495X samples were dialyzed against 50 mM Tris pH 9.5 using a 30 K centricon tube with a nitrocellulose membrane.

### Peptide Synthesis

The FUS RGG peptide corresponding to the C-terminal RGG region (RGG3) (Table 1) was chemically synthesized by the Peptide Synthesis Unit at the University of Pittsburgh Center for Biotechnology and Engineering. The full-length HCV core protein is 191 amino acids long and contains three basic domains (Cristofari et al., 2004; Ivanyi-Nagy et al., 2006). The first two domains, amino acids 2–23 and 38–74, were combined to form a 58 amino acid sequence peptide. This core peptide, named 2BD (Ivanyi-Nagy et al., 2006), was chemically synthesized by University of Pittsburgh Center for Biotechnology and Engineering.

### Polyacrylamide Gel Electrophoresis

20 μM RNA samples were annealed by heating at 95°C in the presence of 25 mM KCl and slowly cooled for 20 min to 25°C. FUS RGG3 was then added in varying ratios and allowed to equilibrate at 25°C for 20 min. Samples were loaded onto a 20% acrylamide:bis-acrylamide gel run at 88 V for ~4 h at 4°C. Gels were visualized by UV-shadowing at 254 nm (Hendry and Hannan, 1996) on an AlphaImager (AlphaInnotech). To test for GQ formation in Shank1a and PSD-95 M1 and M2 mutants, 20% native acrylamide gels were run in a similar manner and subsequently stained in *N*-methyl mesoporphyrin IX (NMM), a GQ-specific stain (Arthanari et al., 1998). The native gel electrophoresis experiments were performed at least in duplicate.

### ^1^H NMR Spectroscopy

G-quadruplex formation was monitored by performing one-dimensional (1D) proton (^1^H) nuclear magnetic resonance (NMR) spectroscopy experiments at 25°C on a 500 MHz Bruker AVANCE spectrometer. Water suppression was performed using the Watergate pulse sequence (Piotto et al., 1992). Samples of each mRNA investigated were prepared in 10 mM cacodylic acid, pH 6.5, in a 90% H_2_O/10% D_2_O ratio to a final volume of 250 μL. Spectra were acquired in the absence and in the presence of 150 mM KCl.

### Circular Dichroism (CD) Spectroscopy

All experiments were performed on a Jasco J-810 spectropolarimeter at 25°C, using a 1 mm path-length quartz cuvette (Starna Cells). 200 μL volumes of 10 μM samples of RNA were prepared in 10 mM cacodylic acid buffer, pH 6.5. GQ formation was monitored between 200 and 350 nm by titrating KCl in the range 0–150 mM, and averaging a series of seven scans with a 1 s response time and a 2 nm bandwidth. The spectra were corrected by subtracting the cacodylic acid buffer contributions.

### UV Spectroscopy Thermal Denaturation

UV thermal denaturation curves were acquired using a Varian Cary 3E UV-visible spectrophotometer with a Peltier temperature control cell holder. Samples were annealed in the presence of 10 mM cacodylic acid, pH 6.5, with 0.5 mM KCl for the PSD-95 GQ2 wild type and mutant M1 and 2.5 mM KCl for the Shank1a GQ wild type and mutant M1, respectively. Two different KCl concentrations were used for these RNAs since the PSD-95 GQ is extremely stable and at 2.5 mM KCl the hypochromic transition corresponding to its GQ dissociation is incomplete (Stefanovic et al., 2015). Two hundred microliter of mineral oil was slowly added on top of the 200 μl samples of 10 μM RNA to prevent evaporation. The RNA samples were heated from 25 to 95°C at a rate of 0.2°C/min, recording the absorbance at 295 nm, wavelength identified as the most sensitive to GQ dissociation (Mergny et al., 1998), at 1°C intervals. The hypochromic transition of the GQ dissociation was identified and fit with equation (1):

(1) $$\begin{array}{l} A\left(T\right)=\frac{A_{U}+A_{F}e^{\frac{-\Delta H^{\circ }}{RT}}e^{\frac{\Delta S^{\circ }}{R}}}{e^{\frac{-\Delta H^{\circ }}{RT}}e^{\frac{\Delta S^{\circ }}{R}}+1} \end{array}$$

where A_U_ and A_F_ represent the absorbance of the unfolded and native GQ structures, respectively, and *R* is the gas constant.

### Steady-State Fluorescence Spectroscopy

Steady-state fluorescence spectroscopy experiments were performed on a Horiba Jobin Yvon Fluoromax-3 with accompanying software fitted with a 150 W ozone-free xenon arc lamp. Experiments were performed in a 150 μl sample volume, 3-mm path-length quartz cuvette (Starna cells). The excitation wavelength for the 2-AP containing samples was set to 310 nm with bandpass excitation and emission of the monochromators set to 5 nm, and the emission recorded between 330 and 450 nm. All binding experiments were performed at 23°C. Increasing concentrations of the FUS RGG3 or full-length FUS were titrated at 30–50 nM increments to a fixed RNA concentration of 150 or 200 nM in 10 mM cacodylic acid, pH 6.5, in the presence of 150 mM KCl. Experiments were performed in the presence of a 5-fold excess BSA or HCV peptide to reduce non-specific binding. Emission values were corrected for free RGG3 and full-length FUS and data was normalized to the free RNA fluorescence intensity at 371 nm. Experiments were performed in triplicate, the intensity was normalized for each experiment and plotted as a function of the peptide or protein concentration and fit to equation (2):

(2) $$\begin{array}{l} F=1+\left(\frac{I_{B}}{I_{F}}-1\right)*\frac{\left(K_{d}+{\left[P\right]}_{t}+{\left[RNA\right]}_{t}\right)-\sqrt{{\left(K_{d}+{\left[P\right]}_{t}+{\left[RNA\right]}_{t}\right)}^{2}-4*{\left[P\right]}_{t}*{\left[RNA\right]}_{t}}}{2*{\left[RNA\right]}_{t}} \end{array}$$

where I_F_ and I_B_ represent steady-state fluorescence intensities of free and bound RNA, respectively. [RNA]_t_ is the total fixed RNA concentration and [P]_t_ is the total protein concentration. The protein-RNA complex dissociation constant, K_d_, was determined for each experiment by fitting the binding curves with equation (2). Reported K_d_ represents an average of the three K_d_ values and the reported error is the standard deviation. The plots shown are representative of the binding curves from one experiment.

### RNA-Based Affinity Pull-Down Assay

The biotinylated PSD-95 Q1-Q2 RNA, biotinylated PSD-95 M2, biotinylated Shank1a GQ or the biotinylated Shank1a M2 probes in 10 mM cacodylic acid pH 6.5 containing 150 mM KCl were denatured at 95°C for 5 min and cooled at room temperature for 15 min. 5 μM of each probe was incubated for 20 min at room temperature with E17 mouse brain lysate obtained by lysing the cells using RIPA buffer (150 mM NaCl, 50 mM Tris-HCl, pH 8.0, 1% NP-40, 0.5% deoxycholate and 0.1% SDS), as well as protease and RNase inhibitors. NeutrAvidin agarose (Thermo Scientific, Waltham, MA) pre-blocked with BSA was used to precipitate the probes. After extensive washing with RIPA buffer, the pellets were prepared for immunoblot with 5x sample buffer. Proteins were detected by immunoblot against FMRP (1:5000, Sigma-Aldrich) and against FUS (1:1000, Santa Cruz Biotechnology) and GAPDH (1:2000, Cell Signaling). The RNA-based affinity pull-down experiments were performed in triplicate.

## Results and Discussion

### FUS C-terminal RGG Motif Recognizes G Quadruplex RNA Sequences Specifically

**Figure 2:**
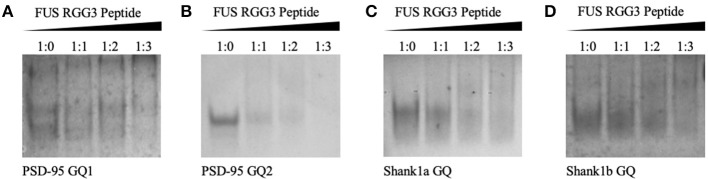
Twenty percent native PAGE, 88 volts, 4°C for 4 h. PSD-95 GQ1 RNA **(A)**, PSD-95 GQ2 RNA **(B)**, Shank1a GQ **(C)**, and Shank1b GQ **(D)** (lane 1) in the presence of increasing FUS RGG3 concentrations (lanes 2–4). Gels were visualized by UV shadowing at 254 nm. These experiments were performed in duplicate and the gels shown are representative results from one experiment.

Since FUS RGG3 was the only FUS motif capable of binding the GQ structure in a study by Takahama et al. (2013), we initially used a peptide composed of the FUS RGG3 domain (amino acids 449–526) and tested its binding to PSD-95 and Shank1 mRNAs. Both PSD-95 mRNA and Shank1 mRNA were shown to form two GQ structures in their 3′-UTR, named PSD-95 GQ1 and PSD-95 GQ2 (Stefanovic et al., 2014), and Shank1a GQ and Shank1b GQ (Zhang et al., 2014). Native polyacrylamide gel electrophoresis (PAGE) was used to test the binding of FUS RGG3 to the GQ structures formed by PSD-95 and Shank1 mRNAs (Figure 2). FUS RGG3 has 19 positively charged amino acids and given the short sequence lengths of the GQs investigated (Table 1), the RNA-peptide complex will have an overall positive charge resulting in blurry, smeared bands on the gel. Thus, the peptide-RNA complex formation was monitored by the disappearance of the free RNA band (full gels shown in Supplemental Figure 1). As seen in Figures 2A–D, the free RNA band (lane 1) diminished when the FUS RGG3 peptide was added in a 1:1, 1:2, and 1:3 ratios (lanes 2–4), indicating the formation of complexes between the FUS RGG3 and the RNA GQs investigated. Since PSD-95 GQ1 is dynamic, forming alternate conformations (Figure 2A, lane 1 and Stefanovic et al., 2014), in this study we only focused on PSD-95 GQ2 (Figure 2B, Stefanovic et al., 2014) since it forms a single conformation (Figure 2B, lane 1). Similarly, from the two GQ structures formed in the Shank1 3′-UTR, in this study we only focused on Shank1a (Figure 2C), as this sequence is conserved in mammals, whereas Shank1b is not (Zhang et al., 2014).

**Figure 3:**
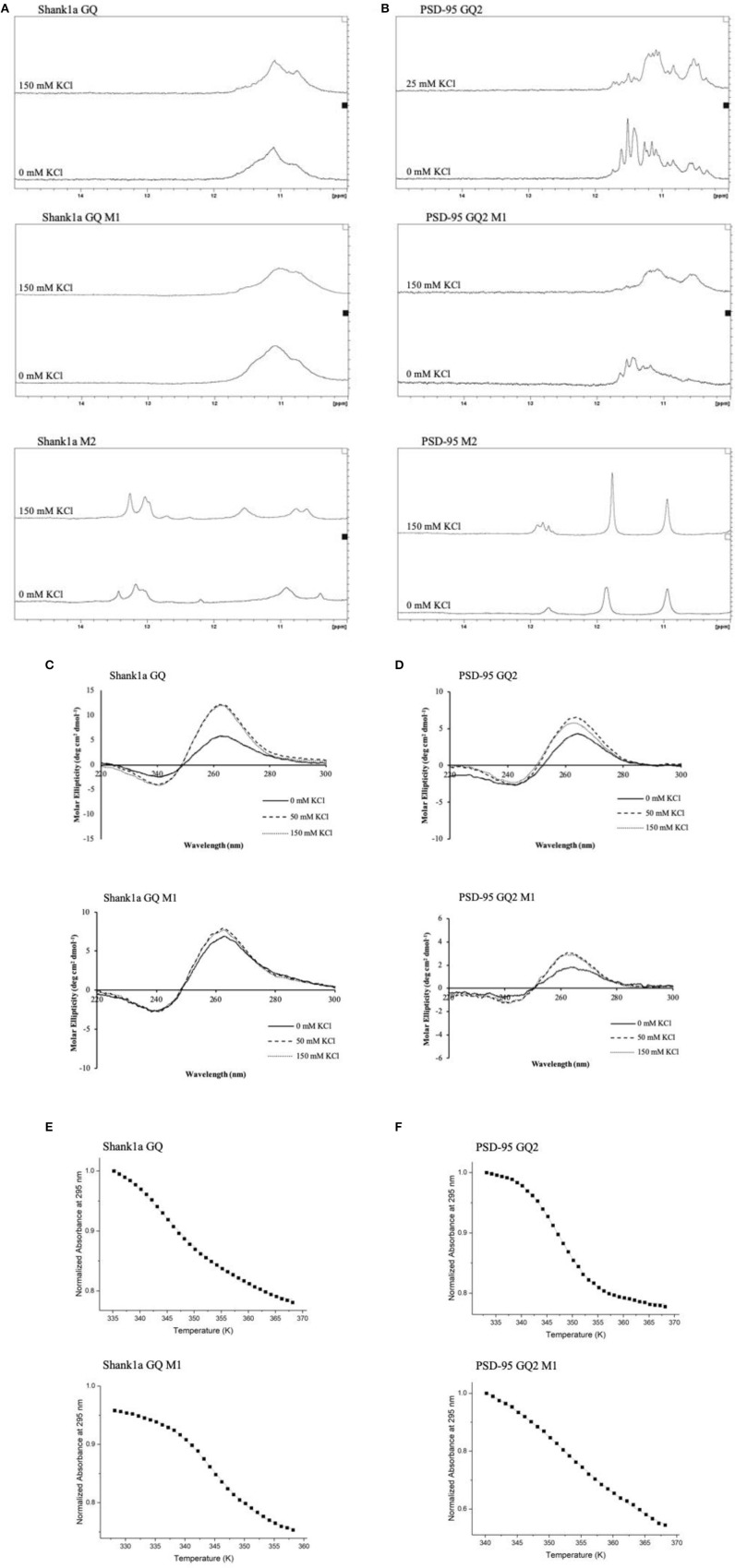
^1^H NMR spectra of Shank1a GQ WT (**A**, top panel), Shank1a GQ M1 (**A**, middle panel), Shank1a M2 (**A**, bottom panel), PSD-95 GQ2 WT (**B**, top panel), PSD-95 GQ2 M1 (**B**, middle panel), and PSD-95 M2 (**B**, bottom panel). Both WT and M1 mutant sequences for Shank1a GQ and PSD-95 GQ2 show imino proton resonances between 10 and 12 ppm indicative of GQ formation, whereas spectra for the M2 mutant sequences do not. CD spectra of Shank1a GQ WT (**C**, top panel), Shank1a GQ M1 (**C**, bottom panel), PSD-95 GQ2 WT (**D**, top panel) and PSD-95 GQ2 M1 (**D**, bottom panel) display a positive band at 265 nm and a negative band at 240 nm, indicating that the parallel GQ fold was maintained for all sequences. UV-thermal denaturation profiles for Shank1a GQ WT (**E**, top panel), Shank1a GQ M1 (**E**, bottom panel), PSD-95 GQ2 WT (**F**, top panel), and PSD-95 GQ2 M1 (**F**, bottom panel). Shank1a GQ mutant was denatured in the presence of 2.5 mM KCl and 10 mM cacodylic acid, pH 6.5. PSD-95 GQ2 mutant was denatured in the presence of 0.5 mM KCl and 10 mM cacodylic acid, pH 6.5. Hypochromic transitions were fit using Equation (1) (Materials and Methods).

FUS was shown to bind telomeric DNA and RNA GQ structures specifically over the single stranded and double helix conformations (Takahama et al., 2013), but up to this point FUS has not been shown to bind GQ structures formed by neuronal mRNAs. However, FUS targets other secondary structures as well, as it has been shown to bind GU rich sequences using its RRM and all three of its RGG domains, with its RGG domain 2 and 3 aiding in E1A mRNA alternative splicing (Lerga et al., 2001; Iko et al., 2004). A hairpin structure with UU or UC as the first base pair in the loop and a conserved GGUG sequence were additionally shown to be targeted by FUS (Hoell et al., 2011). Moreover, it has recently been shown that a FUS fragment spanning its RNA recognition motif (RRM), RGG2 box and Zn finger domain recognizes specifically an NGGU motif within a stem-loop RNA (Loughlin et al., 2019). Though Shank1a GQ and PSD-95 GQ2 sequences do not show evidence of stem-loop formation as no imino proton resonances are present in their ^1^H NMR spectral region 12–14.5 ppm which corresponds to imino protons originating from Watson-Crick base pairs (Figures 3A,B top panels and Stefanovic et al., 2014; Zhang et al., 2014), both RNAs contain the GGUG motif (Figure 1B), which may be recognized by FUS, independent of the GQ structure formation.

To determine if the GQ structure *per se* is sufficient for FUS recognition, we mutated the GGUG stretches to GGCG in both Shank1a GQ and PSD-95 GQ2 to create the Shank1a GQ M1 and PSD-95 GQ2 M1 mutants in such a way that the ability of the mutant sequences to form GQ structures was not impacted (arrows and dashed circles in Figure 1B and Table 1). Additionally, we created two other mutants (named Shank1a M2 and PSD-95 M2) in which both the GGUG stretches were mutated to GGCG and the G quadruplex structures were disrupted by replacing Gs within the G tracts with Cs (Table 1). ^1^H NMR spectroscopy experiments were performed on these four mutants to examine the preservation of the GQ structure in the Shank1a GQ M1 and PSD-95 GQ2 M1 mutants and its disruption in the Shank1a M2 and PSD-95 M2 mutants, by monitoring the imino proton resonance region between 10 and 12 ppm (Fürtig et al., 2003). Spectra were acquired for all mutant RNAs in the absence and presence of 150 mM KCl, as these ions stabilize the GQ structures. As shown in Figures 3A,B middle panels, even in the absence of KCl, resonances were observed in the region between 10 and 12 ppm for both Shank1a GQ M1 and PSD-95 GQ2 M1 RNAs. Upon addition of KCl, these resonances were broadened for both RNAs. This data shows that the Shank1a GQ M1 and PSD-95 GQ2 M1 retain the ability to fold into GQ structures. The Shank1a M2 and PSD-95 M2 RNAs show imino proton resonances in the region 12–14.5 ppm corresponding to Watson-Crick base pairs, but also sharp resonances in the region 10–12 ppm at both 0 and 150 mM KCl (Figures 3A,B, bottom panels). While the presence of broader resonances in the region 10–12 ppm is indicative of G quadruplex structure formation, sharp resonances in the same region have also been observed in structures that do not form such structures, being assigned to imino protons engaged in GU wobble base pairs or non-canonical GG or GA base pairs (Nonin-Lecompte et al., 2001; Bhattacharya et al., 2002). Shank1a M2 and PSD-95 M2 RNAs cannot form GQ structures because they lack stretches of G repeats, however, when folded bimolecularly with the RNA Structure prediction software (Reuter and Matthews, 2010; Supplemental Figure 2) they are predicted to have both Watson-Crick base pairs, GU base pairs, and the potential for GG or GA base pairs. To further confirm that the sharp resonances present in the region 10–12 ppm of the Shank1a M2 and PSD-95 M2 RNAs do not originate from GQ structures, whereas the broad resonances observed in the same region of the Shank1a GQ M1 and PSD-95 GQ2 M1 RNAs originate from GQ structures, we performed native gel electrophoresis in the presence of 25 mM KCl (Supplemental Figure 3). The gels were visualized first by UV shadowing where all RNA bands were visible, followed by staining with NMM, a GQ-specific dye. Both Shank1a GQ M1 and PSD-95 GQ2 M1 RNAs stain in NMM, indicating GQ formation (Supplemental Figure 3A), whereas neither Shank1a M2 nor PSD-95 M2 RNAs stain in NMM indicating that they do not form GQ structures (Supplemental Figure 3B).

Both wild-type Shank1a GQ and PSD-95 GQ2 form parallel GQ structures (Stefanovic et al., 2014; Zhang et al., 2014; Figures 3C,D, top panels), thus, CD spectroscopy experiments were performed in the presence of increasing KCl concentrations to determine if the Shank1a GQ M1 and PSD-95 GQ2 M1 mutants retained the parallel fold. In the absence of KCl, a negative band at 240 nm and a positive band at 265 nm were observed for the Shank1a GQ M1 and PSD-95 GQ2 M1 mutants, indicative of a parallel fold (Figures 3C,D bottom panels; Williamson, 1994; Ðapić, et al., 2003). The band intensity at 265 nm increased upon addition of KCl for both mutants, indicating the further stabilization of their GQ structure by K^+^ ions.

Finally, UV-thermal denaturation experiments were used to compare the wild type and mutant M1 GQ stability for each RNA. Samples of 10 μM RNA in 10 mM cacodylic acid, pH 6.5, were thermally denatured in the presence of 0.5 mM and 2.5 mM KCl for the PSD-95 GQ2 wild type and M1 mutant (Figure 3E and Supplemental Figures 4A,B) and for Shank1a GQ wild type and M1 mutant, respectively (Figure 3F and Supplemental Figures 4C,D), monitoring the absorbance changes at 295 nm, wavelength most sensitive to GQ dissociation (Mergny et al., 1998). The UV thermal denaturation profile for Shank1a GQ wild type and M1 mutant RNAs at 2.5 mM KCl contained a hypochromic transition which was fit with equation (1) to determine a T_m_ of ~73°C for the Shank1a GQ wild type (Figure 3E, top panel), and a T_m_ of ~73°C for Shank1a GQ M1 mutant (Figure 3E, bottom panel). The UV thermal denaturation profile of the PSD-95 GQ2 wild type and M1 mutant RNAs at 0.5 mM KCl contained a hypochromic transition that was fit with equation (1) to determine a T_m_ of ~74°C for PSD-95 GQ2 wild type (Figure 3F, top panel), and a T_m_ of ~80°C for PSD-95 GQ2 M1 mutant (Figure 3F, bottom panel).

Taken together, the NMR spectroscopy, CD spectroscopy, UV spectroscopy and native gel electrophoresis results show that the Shank1a GQ M1 and PSD-95 GQ2 M1 mutant RNAs retain the ability to form parallel GQ structures, whereas these structures are disrupted in the Shank1a M2 and PSD-95 M2 mutants.

**Figure 4:**
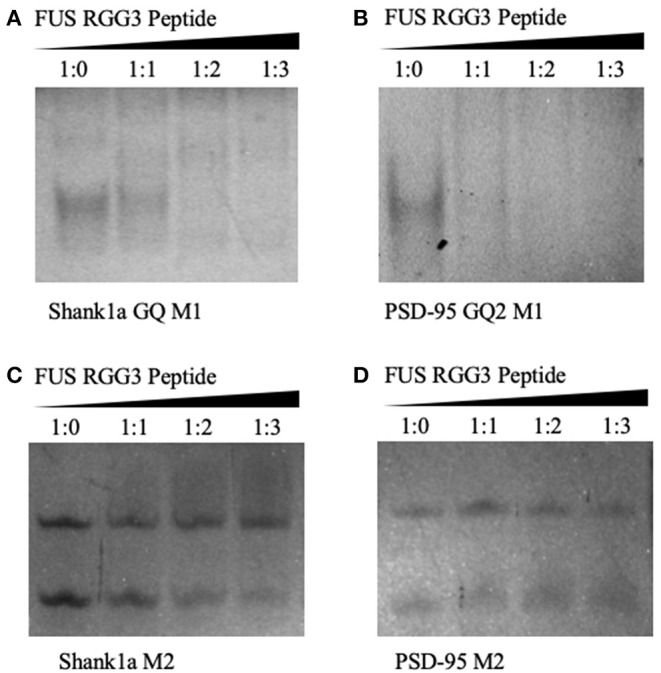
Twenty percent native PAGE, 88 V, 4°C for 4 h. Shank1a GQ M1 **(A)**, PSD-95 GQ2 M1 **(B)**, Shank1a M2 **(C)**, and PSD-95 M2 **(D)** (lane 1) in the presence of increasing FUS RGG3 concentrations (lanes 2–4). Gels were visualized by UV shadowing at 254 nm. These experiments were performed in duplicate and the gels shown are representative results from one experiment.

Native PAGE was next used to test the FUS RGG3 binding to both sets of mutants. As seen in Figures 4A,B both Shank1a GQ M1 and PSD-95 GQ2 M1 RNA mutants, which retain the GQ structures but lack the GGUG recognition motif, are bound by the FUS RGG3 peptide, as the band corresponding to the free RNA (lane 1) diminishes upon addition of FUS RGG3 peptide at a 1:1 ratio (lane 2) and disappears completely at a 1:2 ratio (lane 3; full gels shown in Supplemental Figures 5A,B). Both Shank1a M2 and PSD-95 M2 mutants which lack the GGUG sequence and do not form GQ structures exist in an equilibrium of monomers and dimers (lane 1 in Figures 4C,D; full gel shown in Supplemental Figure 5C). Upon the addition of the FUS RGG3 in increasing ratios, the Shank1a M2 free RNA monomer band decreases slightly in intensity at the higher FUS RGG3 peptide ratios (lanes 3 and 4 in Figure 4C) indicating weak binding. It has been reported previously that FUS has a weakly enriched binding motif containing G/C nucleotides (Colombrita et al., 2012; Ishigaki et al., 2012) and by replacing multiple Gs with Cs to disrupt the GQ formation we have inadvertently created multiple GCC binding sites in the Shank1a M2 mutant. Nonetheless, it should be noted that the free RNA band of the Shank1a GQ wild type and mutant M1 which form GQ structures disappear at higher FUS RGG peptide ratios (compare lanes 3 and 4 in Figures 2C, 4A,C) indicating high affinity for the GQ structures. Taken together, these results indicate that the GQ structure *per se* is sufficient for recognition since both the PSD-95 GQ2 M1 and Shank1a GQ M1 mutants, lacking the GGUG recognition sequence but retaining the GQ structure are bound by the FUS RGG3 peptide, whereas mutants lacking both the GGUG sequence and the GQ structure are either bound with low affinity due to the presence of GCC binding sites (Shank1a M2) or not bound by the FUS RGG3 peptide (PSD-95 M2).

### FUS Has Nanomolar Dissociation Constants for the Neuronal RNA G Quadruplex Structures

**Figure 5:**
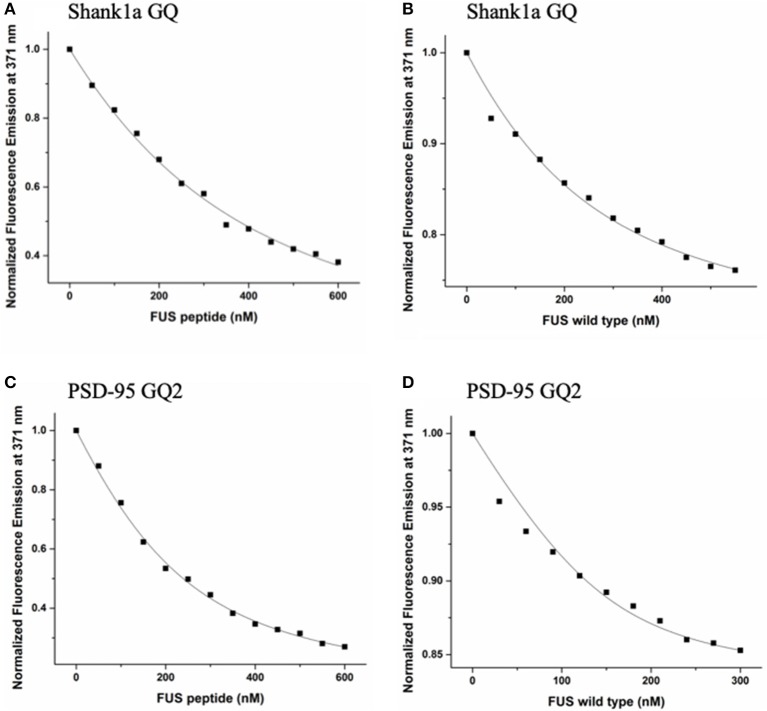
FUS RGG3 was titrated in to 150 nM or 200 nM Shank1a GQ **(A)** and PSD-95 GQ2 **(C)** in the presence of 5-fold excess HCV peptide to prevent non-specific binding. Full-length FUS was titrated into 150 nM or 200 nM Shank1a GQ **(B)** and PSD-95 GQ2 **(D)** in the presence of 5-fold excess BSA to prevent non-specific binding. These experiments were performed in triplicate for each RNA and the dissociation constant, K_d_, was determined for each experiment by fitting the binding curve with Equation (2). The reported K_d_ values represent an average of the three K_d_ measurements and the reported error is the standard deviation. The plots shown are representative of the binding curves from one experiment.

To measure the dissociation constants of the complex formed by FUS with the wild-type PSD-95 GQ2 and Shank1a GQ RNAs, we employed steady-state fluorescence spectroscopy. Single nucleotide 2-AP substitutions were made in the Shank1a GQ and PSD-95 GQ2 at positions 18 and 4, respectively (solid circles in Figure 1B). 2-AP is a highly fluorescent analog of adenine whose steady-state fluorescence is sensitive to its microenvironment (Serrano-Andrés et al., 2006; Bharill et al., 2008), allowing for the monitoring of protein-nucleic acid interactions. Increments of FUS RGG3 or full-length FUS were titrated into annealed samples of 150 or 200 nM PSD-95 GQ2 or Shank1a GQ in 10 mM cacodylic acid, pH 6.5, and 150 mM KCl, while monitoring the changes in steady-state fluorescence of the 2-AP reporter (Figure 5). A 5-fold excess of an unrelated peptide derived from the hepatitis C virus core protein (Ivanyi-Nagy et al., 2006) was used to screen non-specific interactions between FUS RGG3 and RNA.

These experiments were performed in triplicate for each RNA and the dissociation constant, K_d_, was determined for each experiment by fitting the binding curve with Equation (2). The reported K_d_ values represent an average of the three K_d_ measurements and the reported error is the standard deviation. The K_d_ for the Shank1a GQ—FUS RGG3 peptide complex was determined to be 271 ± 21 nM, whereas the K_d_ for the PSD-95 GQ2—FUS RGG3 peptide complex was 92 ± 9 nM (Figures 5A,C). Next, we measured the dissociation constant of the complex formed by the full-length FUS with Shank1a GQ and PSD-95 GQ2. Wild–type FUS was recombinantly expressed using a plasmid encoding for the GST-FUS fusion protein (a kind gift from Dr. Daryl Bosco, Department of Neurology, University of Massachusetts) and purified as described in Kwiatkowski et al. (2009). Similar fluorescence spectroscopy experiments were performed with the full-length FUS in the presence of 5-fold excess BSA to reduce non-specific binding, and the binding curves were fit to equation (2). The K_d_ for the Shank1a GQ—FUS complex had a value of 88 ± 27 nM, whereas that for the PSD-95 GQ2—FUS complex had a value of 28 ±10 nM (Figures 5B,D). Control experiments in which either BSA or GST were titrated showed no change of the steady-state fluorescence of the 2-AP reporters (Supplemental Figure 6), indicating that the changes in fluorescence observed in Figure 5 result from FUS-GQ RNA binding. In both cases, the K_d_ for the full-length FUS binding was lower than that of the isolated FUS RGG3. Thus, it is possible that FUS RGG3 is not solely responsible for binding to the RNA GQ structures, or alternatively, that in the context of the full-length protein, the RGG3 domain might be better oriented allowing it to bind with greater affinity to the GQ structures. In a recent study (Loughlin et al., 2019) it has been shown that the FUS RRM domain bound to several stem-loop RNA structures with K_d_ values ranging from 85 to 130 μM, and that the addition of three RGG repeats to the RRM domain to mimic the FUS RGG2 domain reduced the K_d_ to 10–14 μM. Furthermore, NMR chemical shift perturbation data suggested that in the presence of the RRM domain, which binds specifically to these stem-loop RNAs, the three RGG repeats can remodel the RNA structure. Thus, similarly, it is possible that while the RGG3 domain provides specificity in the recognition of PSD-95 GQ2 and Shank1a GQ RNA structures, the other protein domains reduce the dissociation constant of the full-length FUS-RNA GQ complex by providing additional interactions and/or remodeling the RNA structure. It is important however to note that the dissociation constants we measured for the RGG3 domain or full-length FUS binding to the neuronal mRNA GQ structures are in the low nM range, up to three orders of magnitude less than those measured for the FUS RRM and FUS-RRM-RGG(x3) binding to the stem-loop RNAs, which were in the micromolar range (Loughlin et al., 2019).

**Figure 6:**
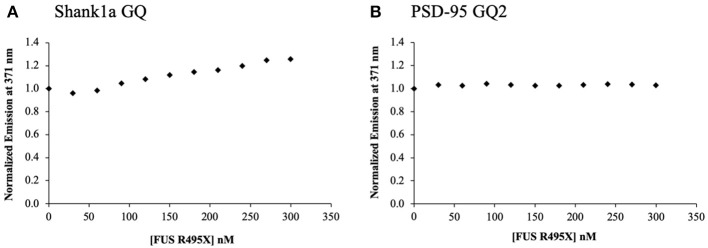
ALS-linked FUS R495X was titrated in 30 nM increments in to 200 nM Shank1a GQ **(A)** and PSD-95 GQ2 **(B)** in the presence of 5-fold excess BSA to prevent non-specific binding. The experiments were performed in triplicate, the curves shown are representative results from one experiment.

In an effort to understand the contribution of the other FUS RNA binding domains to the binding of Shank1a GQ and PSD-95 GQ2, we analyzed the ALS-linked FUS R495X, which lacks the last 32 amino acids at the C-terminus past position 495, and thus, has a truncated RGG3 domain. The FUS R495X has been identified in a family with early-onset ALS (mean age 35 ± 16 years) and has been shown to cause a dramatic increase in cytoplasmic accumulation of FUS compared with other ALS-linked missense mutants (Bosco et al., 2010) and to have an altered association with stress granules (Baron et al., 2013). Full-length FUS R495X was titrated into annealed samples of 200 nM PSD-95 GQ2 or Shank1a GQ in 10 mM cacodylic acid, pH 6.5, and 150 mM KCl, in the presence of an excess of a 5-fold BSA. We observed a linear increase of the steady-state fluorescence of the 2-AP reporter in Shank1a GQ (Figure 6A), indicating low affinity binding, whereas no changes were observed in PSD-95 GQ2 (Figure 6B). Since the 2-AP reporter is located in an eight nucleotide loop containing the GGUG motif within the GQ structure, it is possible that the changes in fluorescence in Shank1a GQ reflect the binding of this motif by FUS R495X. Since the RGG1 and RGG2 domains are intact in FUS495X and this mutant does not bind Shank1a GQ and PSD-95 GQ2 with high affinity like we determined for the wild type FUS, we conclude from these experiments that an intact RGG3 domain is required for the GQ structure recognition.

**Figure 7:**
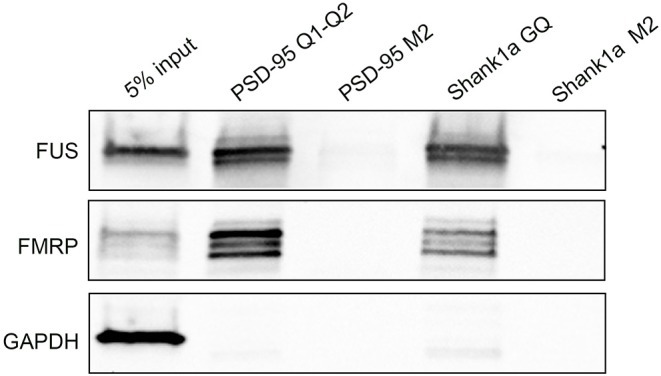
Bi-PSD-95 Q1-Q2, Bi-PSD-95 M2, Bi-Shank1a GQ, and Bi-Shank1a M2 pull down of endogenous FUS and FMRP. The Bi-PSD-95 Q1-Q2, Bi-PSD-95 M2, Bi-Shank1a GQ, and Bi-Shank1a M2 probes were denatured at 95°C in 10 mM cacodylic acid pH 6.5 and 150 mM KCl for 5 min and cooled at room temperature for 15 min. 5 μM of each probe was incubated with E17 mouse brain lysate for 20 min at room temperature and NeutrAvidin agarose (Thermo Scientific, Inc.) pre-blocked with BSA was used to precipitate the probes. After extensive washing, proteins were detected by immunoblot against FUS **(top panel)** and FMRP **(middle panel)**. GADPH **(bottom panel)** was used as a control. The pull down experiments were performed in triplicate, the gels shown are representative results from one experiment.

Next, we tested if Shank1a GQ and PSD-95 GQ2 mRNAs can recognize endogenous FUS by incubating biotinylated RNA probes with mouse E17 brain lysate. We have used for the pull-down a biotinylated probe PSD-95 Q1-Q2, that encompasses both PSD-95 GQ1 and GQ2 sequences that we used previously to pull down FMRP (DeMarco et al., 2019) and a biotinylated Shank1a GQ probe and we detected both FUS and FMRP by immunoblot. Additionally, we have performed pull-down control experiments using the biotinylated Shank1a M2 and PSD-95 M2 mutants which lack the GGUG sequence and do not form GQ structures. As seen in Figure 7 (top panel), both PSD-95 Q1-Q2 and Shank1a GQ probes pulled down endogenous FUS, whereas their M2 mutants did not, indicating that the GQ structure is sufficient for recognition. FUS is present as several bands in pull-down experiments, possibly due to its extensive post-translational modifications (He and Ge, 2017). Both PSD-95 Q1-Q2 and Shank1a GQ probes also pulled down FMRP, consistent with previous results (Zhang et al., 2014; DeMarco et al., 2019), whereas their M2 mutants did not (Figure 7, middle panel). GADPH which was used as a control was not pulled down by any of the probes (Figure 7, bottom panel).

In this study, we demonstrated for the first time that FUS recognizes the GQ structures formed by two neuronal mRNAs, PSD-95 and Shank1, which encode for proteins important for synaptic plasticity and maintenance. Our results expand the class of GQ RNA structures recognized by FUS and provide for the first time quantitative information about FUS's affinity for GQ RNA structures. The dissociation constants we measured for the complexes formed by FUS with PSD-95 GQ2 and Shank1a GQ are in the nanomolar range: 28 ± 10 and 88 ± 27 nM, respectively. Previous studies have shown that FMRP binds neuronal GQ forming mRNAs with nanomolar dissociation constants to regulate their translation (Evans and Mihailescu, 2012; Blice-Baum and Mihailescu, 2014; Zhang et al., 2014; Stefanovic et al., 2015; DeMarco et al., 2019). The dissociation constant of the FMRP—PSD-95 GQ2 complex was 100 ± 17 nM (DeMarco et al., 2019), whereas that of the FMRP—Shank1a GQ complex was 198 ± 28 nM (Zhang et al., 2014), both in the nM range, but higher than the values we measured for the complexes formed by FUS with these respective GQ forming RNAs. Thus, it is feasible that FUS could directly compete with FMRP for binding these neuronal targets and since FMRP is a translation regulator, by displacing FMRP, FUS could indirectly affect the translation of these mRNAs. However, the FUS-FMRP-RNA interactions are more complex, considering that FUS and FMRP interact directly (Blokhuis et al., 2016; He and Ge, 2017). The FMRP tandem Agenet domain is proposed to recognize methylated arginines within the FUS RGG domains, with the deletion of each individual FUS RGG domains resulting in attenuated interactions with FMRP (He and Ge, 2017). Is has also been shown that FUS mutants lacking both RGG2 and RGG3 domains or lacking the RRM domain do not interact with FMRP (Blokhuis et al., 2016). Taken together, these results suggest that the same FUS domains involved in binding FMRP are also involved in binding some of its RNA targets, and this hypothesis is further supported by our results that the FUS RGG3 domain which is implicated in the FMRP Agenet recognition, also binds the neuronal GQ forming mRNA targets.

Several mutations within the FUS RGG3 domain are associated with ALS, and it has been shown that the FUS R514G and R521G mutants interact similarly with FMRP as compared with wild-type FUS (He and Ge, 2017). Another ALS associated FUS mutant, R521C, has comparable interactions with FMRP, however, the FUS R521C mutant affected the FMRP translation regulator function at the synapse, as the synaptic expression of MAP1B mRNA, a well-characterized FMRP target (Lu et al., 2004; Menon et al., 2008), increased significantly when the cells were transfected with the mutant FUS (Blokhuis et al., 2016). These authors suggested that the FUS R521C mutant increases the synaptic MAP1B translation by competing for binding MAP1B mRNA with FMRP, which is a translational repressor. Our results that wild-type FUS and FMRP bind the same two neuronal GQ forming RNA targets with comparable dissociation constants are supportive of such a model. Since we have previously shown that MAP1B mRNA forms a 5′-UTR GQ structure recognized by FMRP (Menon et al., 2008), we tested by native PAGE if this GQ structure is also bound by the FUS RGG3 domain. As seen in Supplemental Figure 7, the FUS RGG3 domain binds the MAP1B 5′-UTR GQ sequence, this result providing additional support of a direct competition between FMRP and FUS for binding neuronal GQ forming mRNAs.

The results of this study should motivate further studies to fully elucidate the role of the FMRP—FUS—neuronal GQ forming mRNAs interactions in regulating translation. Moreover, given that a number of ALS-associated FUS mutants are located within its RGG3 domain, their neuronal GQ RNA binding properties should also be investigated to increase our understanding of the role of FMRP in the etiology of ALS. Our findings that FUS R495X has impaired binding of Shank1a GQ and PSD-95 GQ2 mRNAs have implications for a potential FUS loss of function at the synapse with respect to the regulation of neuronal mRNAs targets containing GQ structures within their 3′-UTR.

## Data Availability Statement

The datasets generated for this study are available on request to the corresponding author.

## Author Contributions

DM, JI, AV-S, GB, and MM conceived the study, designed the experiments, and analyzed the data. DM, JI, and AV-S performed the experiments. DM, JI, and MM wrote the paper.

### Conflict of Interest

The authors declare that the research was conducted in the absence of any commercial or financial relationships that could be construed as a potential conflict of interest.
