# Supplementary material for: FUS Recognizes G Quadruplex Structures Within Neuronal mRNAs
Source: Front Mol Biosci. 2020 Feb 7;7:6. doi: 10.3389/fmolb.2020.00006 (PMC7018707; doi:10.3389/fmolb.2020.00006)
Supplement: Supplementary file 1 [file Data_Sheet_1.docx]

**Supplemental Figure 1.** 20% native PAGE full FUS RGG3 binding gels of PSD-95 GQ1 **(A)**, PSD-95 GQ2 **(B)**, Shank1a GQ **(C)**, and Shank1b GQ **(D)**.

**Supplemental Figure 2.** Predicted bimolecular structures for Shank1a M2 (A) and PSD-95 M2 (B) using the RNA Structure software.

**Supplemental Figure 3.** 20% native PAGE gel demonstrating the formation of GQ structures within Shank1a GQ M1 and PSD-95 GQ2 M1 **(A)** using a GQ-specific stain, NMM. Conversely, Shank1a M2 and PSD-95 M2 do not stain **(B)**, indicating GQ structures do not form. A 26-nucleotide RNA sequence previously characterized in our lab to form a GQ structure (5’-GGGACGGGGAAUGGGAGGGGUAGAUU-3’) was used as a positive GQ control, whereas a 90-nucleotide hairpin RNA (5’-GGCCGGCGCCCGAGCU CUGGCUCCGUGUCUUCACUCC CGUGCUUGUCCGAGGAGCGAGAGAGAGACGGAGGCUGUGCUGGGGCAGCUGGA-3’) was used as the negative GQ control. Note the formation of dimers in both Shank1a M2 and PSD-95 M2, as predicted by the RNA Structure software (Supplemental Figure 2).

**Supplemental Figure 4.** Full UV-thermal denaturation spectra of Figures 3E and 3F for the Shank1a GQ **(A)**, Shank1a GQ M1 **(B)**, PSD-95 GQ2 **(C)**, and PSD-95 GQ2 M1 **(D)**. Shank1a GQ and Shank1a GQ M1 were denatured in the presence of 2.5 mM KCl and 10 mM cacodylic acid, pH 6.5. PSD-95 GQ2 and PSD-95 GQ2 M1 were denatured in the presence of 0.5 mM KCl and 10 mM cacodylic acid, pH 6.5.

**Supplemental Figure 5.** 20% native PAGE full FUS RGG3 binding gels of Shank1a GQ M1 **(A)**, PSD-95 GQ2 M1 **(B)**, and Shank1 M2 / PSD-95 M2 **(C)**.

**Supplemental Figure 6.** Fluorescence spectroscopy experiments showing the results of BSA titration into 200 nM Shank1a GQ **(A)** and PSD-95 GQ2 **(B)** and of GST titration into 200 nM Shank1a GQ **(C)** and PSD-95 GQ2 **(D)**.

**Supplemental Figure 7.** 20% native PAGE showing that the FUS RGG3 domain is capable of binding the 34-nucleotide MAP1B 5’-UTR GQ sequence **(A)**. The MAP1B 5’-UTR sequence used was 5’-GGCGCUGGGAGAGGGCGGAGGGGGAGGCGGCGCC-3’ (Menon et al. 2008). Full FUS RGG3 binding gel of MAP1B is shown in **(B)**.

**Supplemental Figure 1**


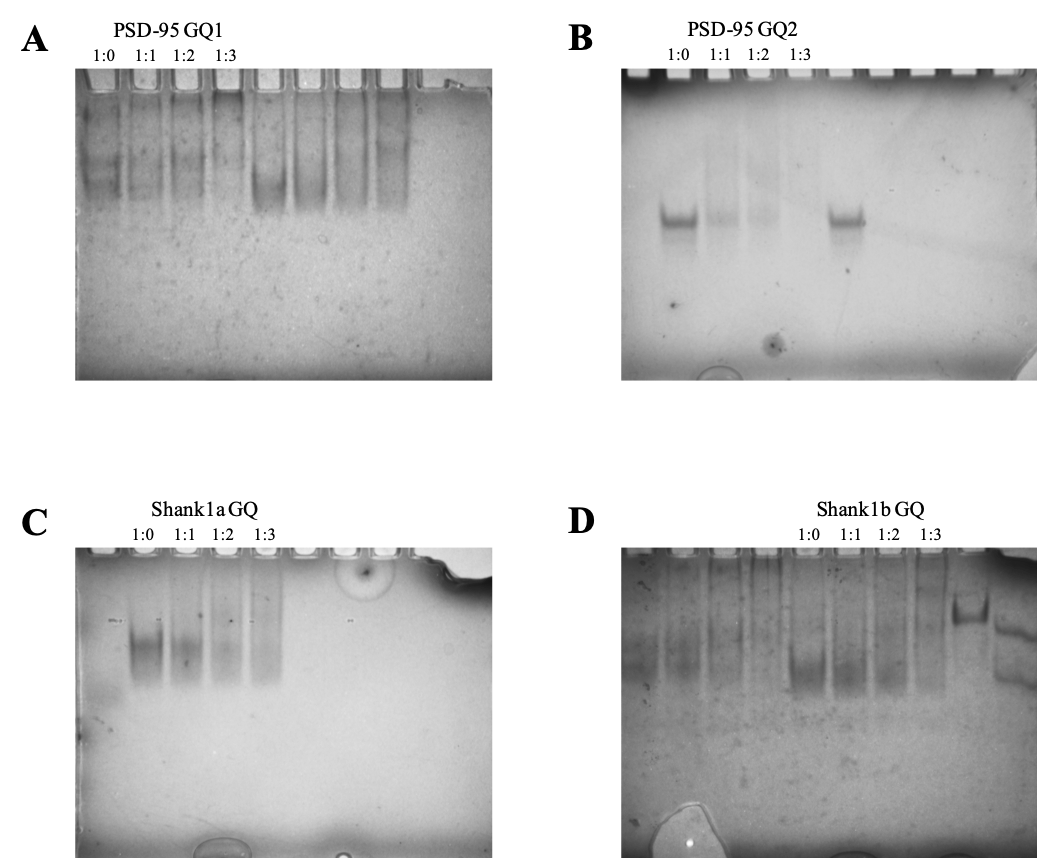


**Supplemental Figure 2**

**
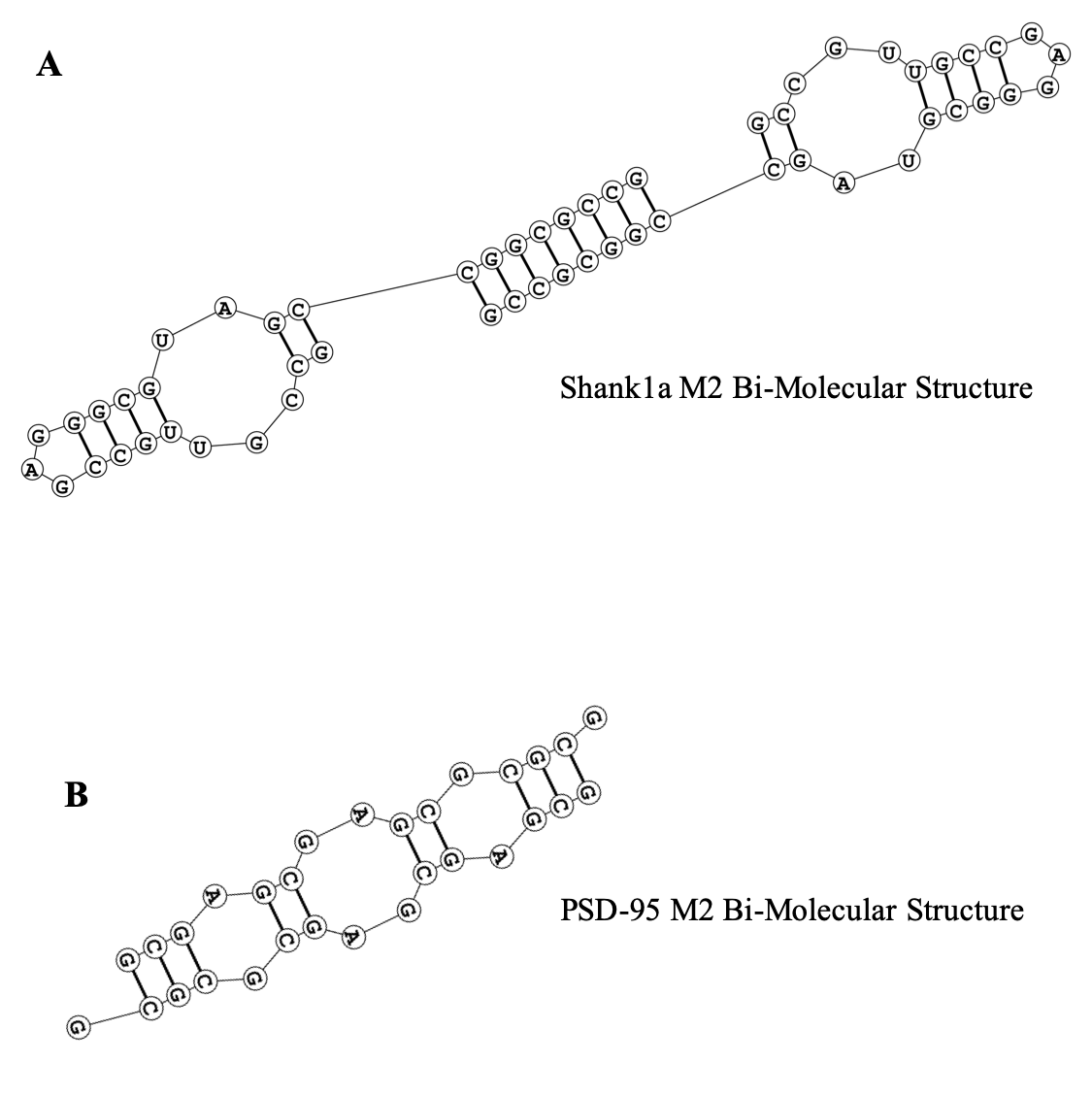
**

**Supplemental Figure 3**


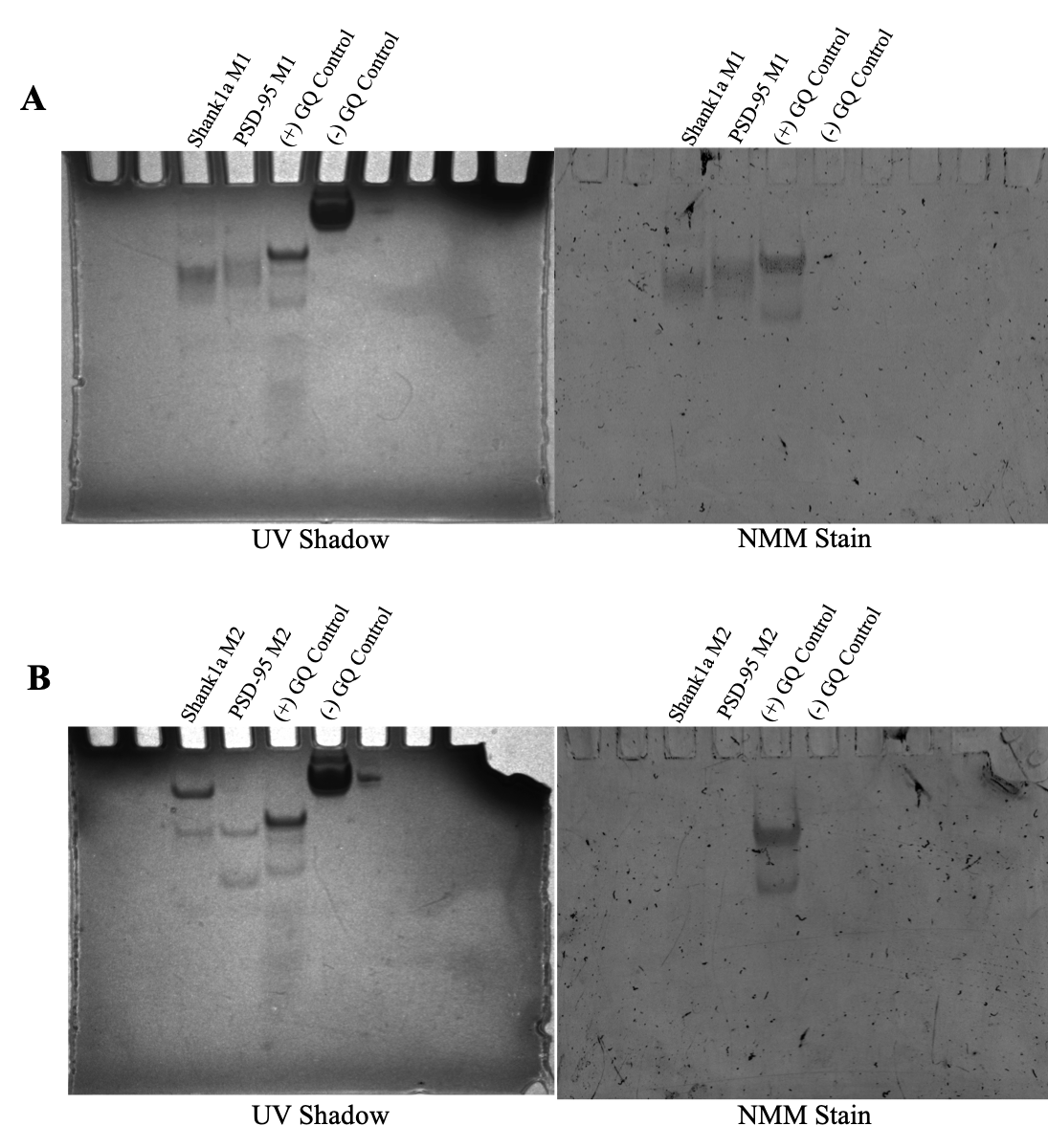


**Supplemental Figure 4**


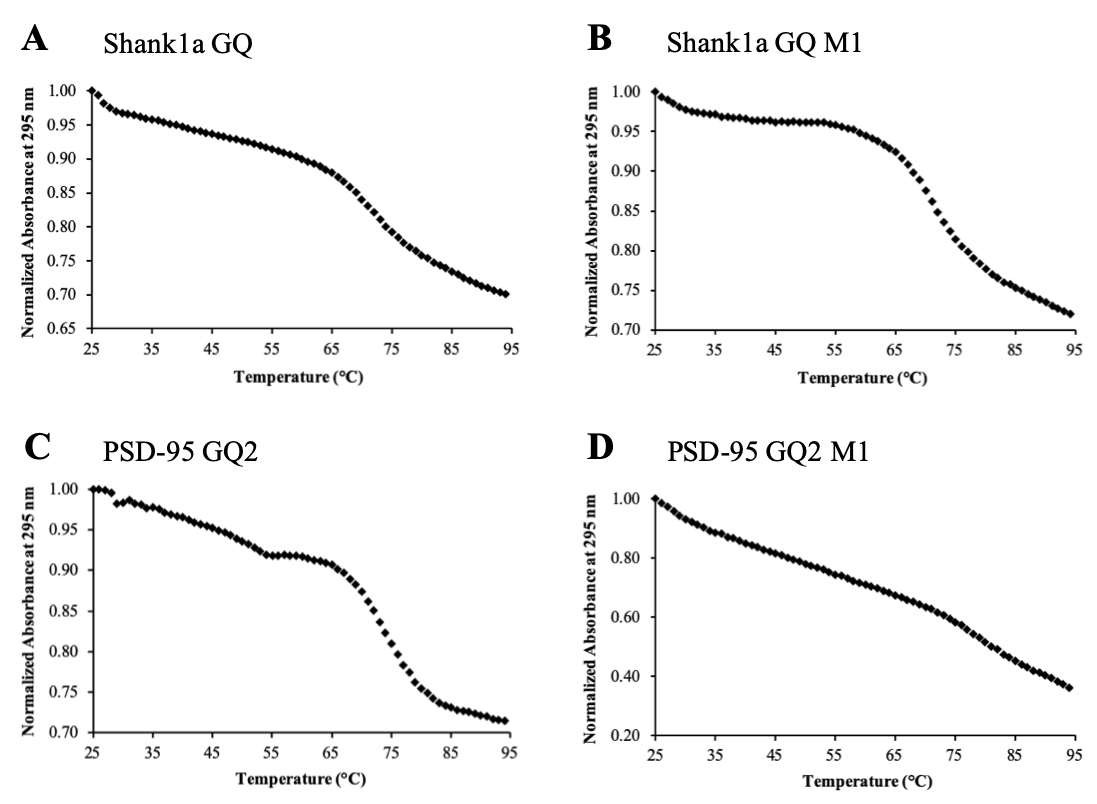


**Supplemental Figure 5**


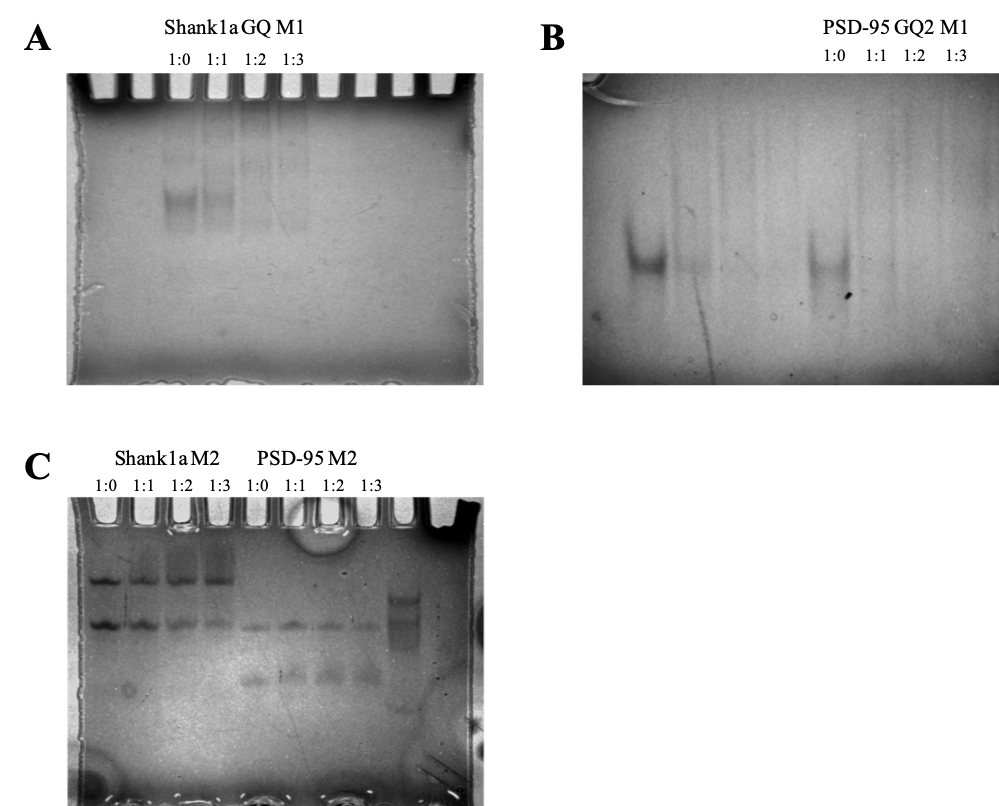


**Supplemental Figure 6**


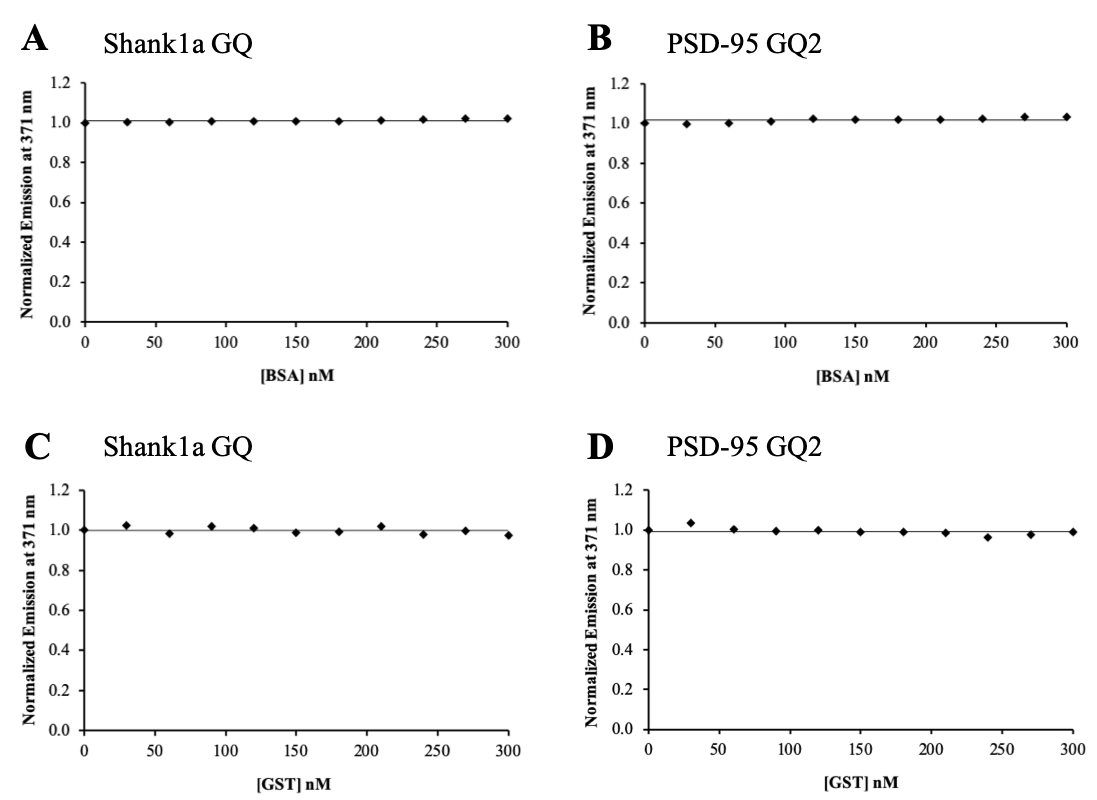


**Supplemental Figure 7**

**
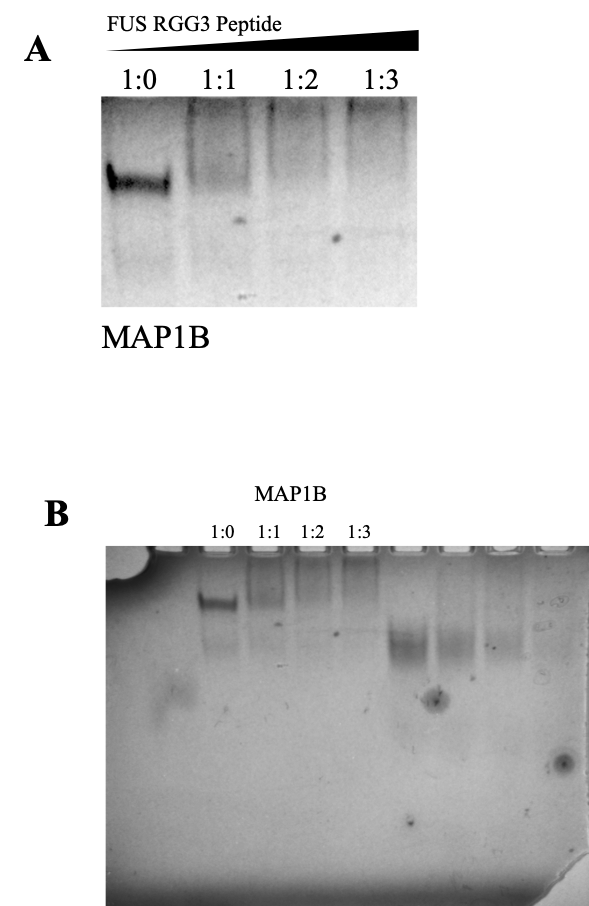
**
